# Familial dysalbuminaemic hyperthyroxinaemia interferes with current free thyroid hormone immunoassay methods

**DOI:** 10.1530/EJE-19-1021

**Published:** 2020-03-26

**Authors:** Serena Khoo, Greta Lyons, Anne McGowan, Mark Gurnell, Susan Oddy, W Edward Visser, Sjoerd van den Berg, David Halsall, Kevin Taylor, Krishna Chatterjee, Carla Moran

**Affiliations:** 1 University of Cambridge Metabolic Research Laboratories, Wellcome-MRC Institute of Metabolic Science, University of Cambridge, Cambridge, UK; 2 Department of Clinical Biochemistry, Addenbrooke’s Hospital, Cambridge, UK; 3 Department of Internal Medicine, Academic Center for Thyroid Diseases, Erasmus MC, Rotterdam, Netherlands; 4 Department of Clinical Chemistry, Erasmus MC, Rotterdam, Netherlands

## Abstract

**Objective:**

Familial dysalbuminaemic hyperthyroxinaemia (FDH), most commonly due to an Arginine to Histidine mutation at residue 218 (R218H) in the albumin gene, causes artefactual elevation of free thyroid hormones in euthyroid individuals. We have evaluated the susceptibility of most current free thyroid hormone immunoassay methods used in the United Kingdom, Europe and Far East to interference by R218H FDH.

**Methods:**

Different, one- and two-step immunoassay methods were tested, measuring free T4 (FT4) and free T3 (FT3) in 37 individuals with genetically proven R218H FDH.

**Results:**

With the exception of Ortho VITROS, FT4 measurements were raised in all assays, with greatest to lowest susceptibility to interference being Beckman ACCESS > Roche ELECSYS > FUJIREBIO Lumipulse > Siemens CENTAUR > Abbott ARCHITECT > Perkin-Elmer DELFIA. Five different assays recorded high FT3 levels, with the Siemens CENTAUR method measuring high FT3 values in up to 30% of cases. However, depending on the assay method, FT4 measurements were unexpectedly normal in some, genetically confirmed, affected relatives of index FDH cases.

**Conclusions:**

All FT4 immunoassays evaluated are prone to interference by R218H FDH, with their varying susceptibility not being related to assay architecture but likely due to differing assay conditions or buffer composition. Added susceptibility of many FT3 assays to measurement interference, resulting in high FT4 and FT3 with non-suppressed TSH levels, raises the possibility of R218H FDH being misdiagnosed as resistance to thyroid hormone beta or TSH-secreting pituitary tumour, potentially leading to unnecessary investigation and inappropriate treatment.

## Introduction

Familial dysalbuminaemic hyperthyroxinaemia, a dominantly inherited condition due to mutant circulating albumin with altered binding affinity for thyroid hormones (TH), is a recognized cause of artefactual elevation in free TH measurements in euthyroid individuals. Following its recognition as a biochemical entity (1, 2), two groups described the molecular basis of FDH, identifying a heterozygous mutation in the albumin gene, changing arginine at codon 218 in the protein to histidine (R218H) (3, 4). The R218H albumin variant is the commonest form of FDH, with a reported prevalence of 1:10 000 in Caucasians (5), but may occur at a frequency of 1 in 100 in Hispanics (6).

In R218H FDH, total thyroxine (T4) levels are characteristically two- to three-fold elevated (3) , with normal or mildly raised total triiodothyronine (T3) concentrations (7), reflecting a ten-fold higher T4 binding affinity (3) and five-fold higher T3 binding affinity of mutant albumin (8). Most clinical laboratories measure free thyroid hormones using automated immunometric assays, recording artefactually high FT4 measurements in this disorder (9, 10). One-step competitive assays are based on the principle that both endogenous free T4 and labelled T4 analogue (assay tracer) compete for binding to an immobilized T4-specific antibody, with measured FT4 concentrations being inversely proportional to the amount of captured labelled tracer. However, in FDH, enhanced interaction of the labelled T4 analogue with mutant albumin decreases its availability to compete with free T4 for capture antibody binding sites, resulting in spuriously high FT4 measurements (9). In contrast, in two-step assays, the labelled T4 analogue tracer is only introduced after an initial step in which the capture antibody and endogenous free T4 have been allowed to equilibrate, followed by a wash step which removes the interfering mutant albumin, purportedly eliminating susceptibility to assay interference. Circulating FT4 measured by ‘gold standard’ assays such as equilibrium or symmetric dialysis or ultrafiltration would typically but not invariably (11) yield normal results, but these methods are labour intensive, time consuming and expensive.

The susceptibility of current FT4 and FT3 immunoassay methods to interference by FDH is unknown. Here, in an unselected cohort of individuals with differing normal hypothalamic-pituitary-thyroid axis setpoints, all harbouring the common R218H albumin variant causing FDH, we have measured FT4 and FT3 levels using most immunoassay methods currently in use in the United Kingdom, Europe and Far East.

## Subjects and methods

### FDH cohort

Serum samples from thirty-seven different, unselected cases of FDH, all harbouring an arginine to histidine mutation at codon 218 (R218H) in the albumin gene, were analysed. Individuals with abnormal serum TSH concentrations, suggesting coexistent thyroid dysfunction, were excluded. Investigations were either clinically indicated or undertaken as part of a protocol approved by our Research Ethics Committee (LREC 98/154) with prior informed consent of participants.

### Free and total thyroid hormone measurements

FT4 and FT3 measurements were undertaken as specified by manufacturers as follows: One-step immunoassay methods used: ADVIA CENTAUR XP^®^: Siemens Medical Solutions Diagnostics (reference range (RR) for FT4: 10.0–19.8 pmol/L and FT3: 3.5–6.5 pmol/L); ELECSYS E170: Roche Diagnostics (RR for FT4: 12.0–22.0 pmol/L and FT3: 3.1–6.8 pmol/L) and VITROS Eci: Ortho Clinical Diagnostics (RR for FT4: 8.9–20.3 pmol/L and FT3: 4.1–7.9 pmol/L). LUMIPULSE: Fujirebio (RR for FT4: 13.5–24.0 pmol/L and FT3: 2.7–6.7 pmol/L). Two-step immunoassay methods used: Wallac DELFIA: Perkin Elmer (RR for FT4: 9.0–20 pmol/L and FT3: 3.5–7.5 pmol/L); ARCHITECT c800: Abbott Ltd Diagnostics (RR for FT4: 8.0–21.0 pmol/L and FT3: 3.8–6.0 pmol/L) and ACCESS 2: Beckman Coulter. (RR for FT4: 6.3–14.0 pmol/L). FT4 measurements by three methods (Ortho VITROS Ec1, Fujirebio LUMIPULSE, Roche ELECSYS E170) were repeated in assay buffer supplemented with 2.5 mM sodium chloride.

### Provenance of assays in National External Quality Assurance Scheme

The UK National External Quality Assurance Scheme (NEQAS) is a programme in which participating laboratories assess consistency and analytical bias of their local assay methods by periodically undertaking biochemical measurements in a panel of reference samples. To assess the range of free thyroid hormone immunoassay methods used in the United Kingdom, we quantified the provenance of FT4 and FT3 measurement methods used by different centres participating in the Quality Assurance Scheme for Thyroid Hormones.

### Statistical analysis

For each assay method, measured mean FT4 and FT3 concentrations were compared to the mean of the manufacturer’s or centre’s reference range and the differences between these means were analysed using a one sample *t*-test, with a *P* value of <0.05 denoting statistical significance.

## Results

**Figure 1:**
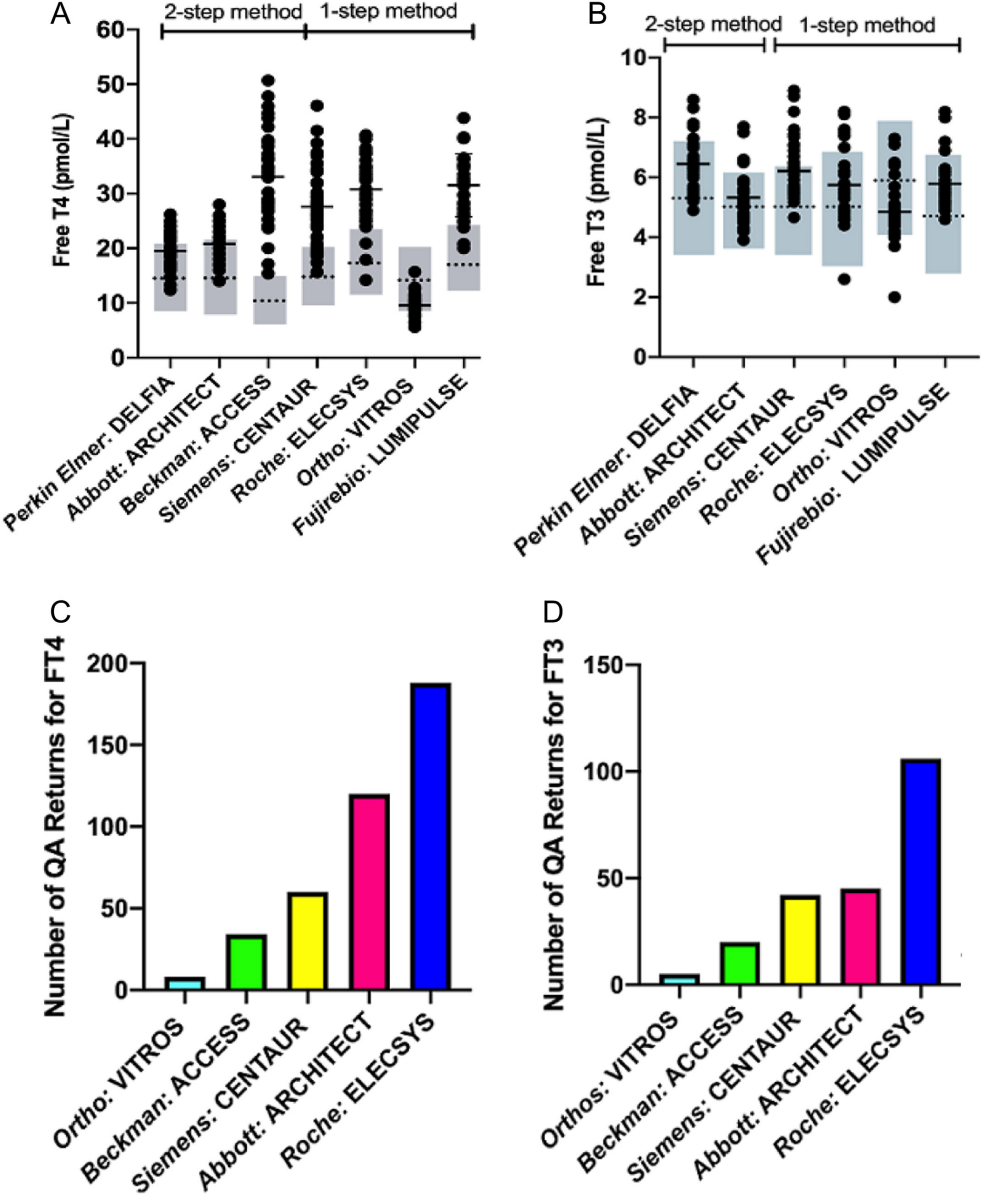
Free T4 (panel A) or Free T3 (panel B) measurements in FDH individuals using different assay methods. Closed circles denote individual data, with horizontal lines corresponding to the mean of measured values. The dotted line and shaded grey box represent the mean and reference range of each assay. (C and D) Histograms showing the number of different FT4 (panel C) or FT3 (panel D) measurement methods recorded by centres returning data in the UK National External Quality Assurance (QA) Scheme.

With the exception of Ortho VITROS, raised FT4 values were recorded in 43% to 100% of FDH cases, depending on the immunoassay method (Table 1). FT4 measurements were all above the upper limit of normal with a two-step assay (Beckman: ACCESS); conversely, in a one-step method (Ortho: VITROS), normal or even underestimated, low FT4 values were recorded (Fig. 1A). Mean measured vs mean of reference range FT4 values were significantly different for all assays, but some two-step methods (Perkin-Elmer: DELFIA; Abbott: ARCHITECT) recorded values closer to the reference range mean than one-step methods (Siemens: CENTAUR; Roche: ELECSYS) (Supplementary Fig. 1A, see section on supplementary materials given at the end of this article). The rank order of deviation in measured vs reference FT4 values with different assay methods (greatest to lowest) was Beckman: ACCESS > Roche: ELECSYS > Fujirebio: LUMIPULSE > Siemens: CENTAUR > Abbott*:* ARCHITECT > Perkin-Elmer: DELFIA. The Ortho VITROS method recorded lower mean FT4 values than its assay reference mean (Supplementary Fig. 1A). Increasing chloride concentration of assay buffer raised FT4 values measured by the Ortho VITROS method markedly, but had no effect on FT4 values measured by the Roche ELECSYS or Fujirebio LUMIPULSE methods (Supplementary Fig. 2).

**Table 1:** Percentage of Free T4 and Free T3 values above the upper limit of reference range using different assay methods.

	Assay method						
	Delfia	Architect	Access	Centaur	Elecsys	Vitros	Lumipulse
Free T4 (%)	46	43	100	84	92	0	92
Free T3 (%)	14	14	–	30	19	0	11

Free T3 measurements were raised (Fig. 1B), but to a lesser degree than FT4, with high values being recorded in 11% to 30% of FDH cases using five different assay methods (Table 1). Differences between measured and reference range mean Free T3 values were smaller, with their rank order (greatest to lowest) being: Siemens CENTAUR > Fujirebio LUMIPULSE > Perkin-Elmer DELFIA > Roche ELECSYS > Abbott ARCHITECT, with the Ortho VITROS method recording mean FT3 values below its assay reference mean (Supplementary Fig. 1B).

We measured FT4 levels in eight individuals with genetically proven R218H FDH from four unrelated families using different assay methods. Unexpectedly, although the index FDH patient from a family exhibited raised FT4 levels, many measurement methods (e.g. Perkin-*Elmer* DELFIA, Abbott ARCHITECT, Siemens CENTAUR) recorded normal FT4 levels in their relatives also known to be affected by this condition (Supplementary Fig. 3).

## Discussion

Using a cohort of unselected, genetically confirmed, cases of familial dysalbuminaemic hyperthyroxinaemia due to the commonest causal albumin variant (R218H) in Caucasian populations, we have systematically evaluated susceptibility to assay interference of most free thyroid hormone measurement methods currently used in the United Kingdom, Europe and Far East. Both one-step (e.g. Roche ELECSYS, Siemens CENTAUR) and two-step (e.g. Beckman ACCESS) FT4 measurement methods are markedly susceptible to interference. In addition, we find that one-step (e.g. Siemens CENTAUR, Roche ELECSYS) and two-step (e.g. Abbott ARCHITECT, Perkin-Elmer DELFIA) FT3 measurement methods are also susceptible to interference, recording high FT3 values in up to 30% of cases. Measurement methods most susceptible to interference (FT4: Roche ELECSYS, Siemens CENTAUR, Beckman ACCESS; FT3: Siemens CENTAUR, Roche ELECSYS, Abbott ARCHITECT) are also the most frequent types of assay participating in the UK national quality assurance scheme (Fig. 1C and D) or are an assay method (Fujirebio LUMIPULSE) that is widely used in the Far East, suggesting that the potential for recording artefactually elevated free thyroid hormone levels and consequent misdiagnosis is of immense clinical relevance.

The varying susceptibility of different measurement methods to interference by FDH does suggest that differing assay methodologies account for this, but our results indicate that one-step vs two-step assay architecture is unlikely to be the major determinant. Thus, a two-step FT4 assay (Beckman ACCESS) recorded highest FT4 values, whereas a one-step FT4 method (Ortho VITROS) recorded normal or even slightly low FT4 values. Indeed, in our experience, discordance between low FT4 measurements in Ortho VITROS and high FT4 values using other methods is highly suggestive of FDH. Our findings accord with previous observations showing that some two-step assays yield spuriously high FT4 values in FDH (10). Manufacturers have sought to minimize susceptibility to measurement interference by altering the composition of incubation buffer to inhibit binding of T4 or labelled T4 analogues (used in one-step but also two-step assays) to albumin. Increasing the concentration of chloride ions in assay buffer has been shown to increase FT4 values even when measured using ‘gold standard’ methods such as equilibrium dialysis (12) or symmetric dialysis (13), with this effect being much more pronounced with FDH vs normalbuminaemic serum samples. Thus, as others have noted previously, the FT4 assay method we found most susceptible to interference (Beckman ACCESS) uses incubation buffer with a high chloride content, whereas the method least susceptible to positive interference (Ortho VITROS) makes measurements using chloride-free medium (13). Supporting this notion, we found that increasing chloride concentration of assay buffer affected FT4 measured by Ortho VITROS markedly. However, increased chloride had minimal effect on FT4 measured by Roche ELECSYS or Fujirebio LUMIPULSE, suggesting that other proprietary constituents of assay buffers, whose identity is difficult to ascertain, are responsible for assay interference in different measurement methods. Moreover, we have found that the current Siemens CENTAUR FT4 measurement method is much more prone to interference by FDH than the assay version we evaluated in 2009 (14), indicating that change in assay design by manufacturers is a further variable influencing susceptibility to interference.

Studies in our large R218H FDH cohort indicate that current FT3 measurement methods are also susceptible to interference, albeit to a lesser degree, possibly also due to the lower T3 binding affinity of mutant albumin. Such artefactual FT3 elevation, generating a pattern of thyroid function tests (raised FT4 and FT3, non-suppressed TSH) which mimics disorders such as Resistance to Thyroid Hormone beta or pituitary, TSH-secreting tumour, is clinically significant because it may provoke unwarranted investigation (e.g. MRI scan, dynamic endocrine testing) or treatment (e.g. pituitary surgery, triiodothyroacetic acid or somatostatin analogue therapy), with attendant cost or morbidity (15). Conversely, low, recorded FT4 levels, measured by the Ortho VITROS method, could lead to a misdiagnosis of central hypothyroidism.

Substantial interindividual differences in free thyroid hormone levels suggest differing setpoints of the hypothalamic-pituitary-thyroid axis in healthy subjects, with a complex, underlying polygenic basis (16). We suggest that such interindividual variation in pituitary-thyroid axis setpoint might also account for normal FT4 values being recorded in some FDH family members even though the index case in the kindred was identified on the basis of raised FT4 using the same measurement method. Accordingly, we suggest that definitive diagnostic methods (e.g. radiolabelled T4 binding to serum proteins, albumin gene sequencing) rather than biochemical screening using free thyroid hormone measurements should be used to ascertain the status of relatives in families with FDH.

The relatively high prevalence and familial nature of FDH, together with susceptibility of both commonly used current FT4 and FT3 immunoassay methods to interference from this condition, suggests that this entity is not only a significant cause of discordant thyroid function but potentially responsible for substantial misdiagnosis, unnecessary investigation and inappropriate management.

## Supplementary Material

Supplementary Figure 1. Each bar denotes the difference between measured mean and assay reference range mean for FT4 (left, panel A) or FT3 (right, panel B) for a particular measurement method. The numerical difference between measured and assay reference range means is shown above each bar. Asterisks denote statistically significant differences in means with a P value of <0.001.

Supplementary Figure 2. Scatter plots showing FT4 measurements in assay buffer with (Y axis) or without (X axis) chloride supplementation using Ortho VITROS (panel A) or Roche ELECSYS (panel B) or Fujirebio LUMIPULSE (panel C) methods. The blue line represents correlation between measurements calculated by Passing-Bablok regression analysis. The line of identical fit is shown in grey.

Supplementary Figure 3. Free T4, expressed as % upper limit of the reference range, in 8 individuals with FDH from 4 unrelated families. Each bar represents an affected individual and each colour represents a different family. ---- denotes upper limit of reference ranges.

## Declaration of interest

W Edward Visser is on the editorial board of EJE. W Edward Visser was not involved in the review or editorial process for this paper, on which he is listed as an author. The other authors have nothing to disclose.

## Funding

Our research is supported by funding from the Wellcome Trust (210755/Z/18/Z to K C) and NIHR Cambridge Biomedical Research Centre (C M, M G and K C).

## Author contribution statement

S K, G L, A M, S O, S B and K T performed data collection. M G, W E V, D H, K C and C M contributed to the interpretation, analysis and writing of this manuscript. K C and C M are co-equal senior authors.
