# Supplementary figures and images for: Familial dysalbuminaemic hyperthyroxinaemia interferes with current free thyroid hormone immunoassay methods
Source: Eur J Endocrinol. 2020 Mar 26;182(6):533–8. doi: 10.1530/EJE-19-1021 (PMC7222281; doi:10.1530/EJE-19-1021)

A

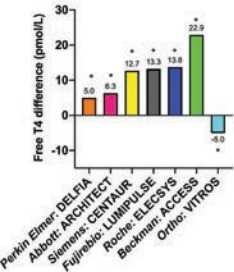

B

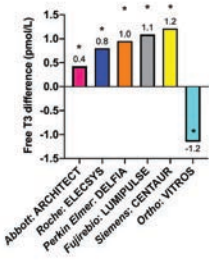

Supplement: Supplementary Figure 1. Each bar denotes the difference between measured mean and assay reference range mean for FT4 (left, panel A) or FT3 (right, panel B) for a particular measurement method. The numerical difference between measured and assay reference range means is shown above each bar. Asteris [file supplementary_figure_1.pdf]

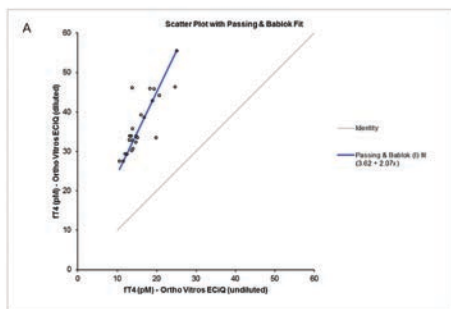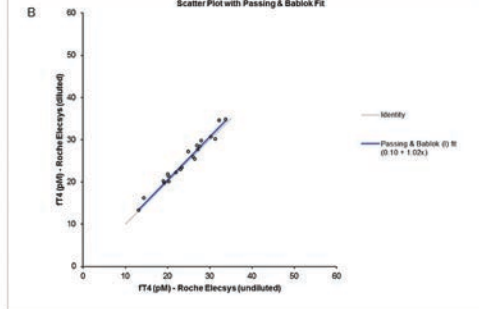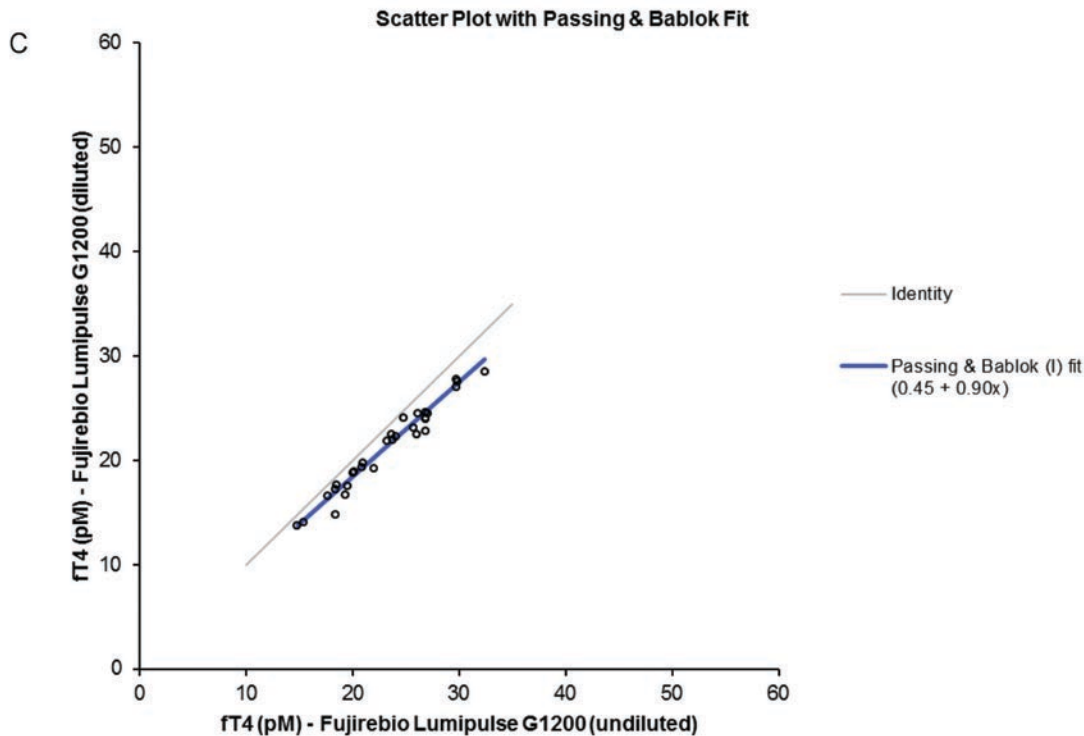

Supplement: Supplementary Figure 2. Scatter plots showing FT4 measurements in assay buffer with (Y axis) or without (X axis) chloride supplementation using Ortho VITROS (panel A) or Roche ELECSYS (panel B) or Fujirebio LUMIPULSE (panel C) methods. The blue line represents correlation between measurements calcul [file supplementary_figure_2.pdf]

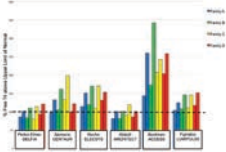

Supplement: Supplementary Figure 3. Free T4, expressed as % upper limit of the reference range, in 8 individuals with FDH from 4 unrelated families. Each bar represents an affected individual and each colour represents a different family. ---- denotes upper limit of reference ranges. [file supplementary_figure_3.pdf]
